# Identification of Insecticidal Constituents of the Essential Oil of *Curcuma wenyujin* Rhizomes Active against *Liposcelis bostrychophila* Badonnel

**DOI:** 10.3390/molecules171012049

**Published:** 2012-10-15

**Authors:** Zhi Long Liu, Na Na Zhao, Chun Ming Liu, Ligang Zhou, Shu Shan Du

**Affiliations:** 1 Department of Entomology, China Agricultural University, Haidian District, Beijing 100193, China; 2 Department of Plant Pathology, China Agricultural University, Haidian District, Beijing 100193, China; 3 College of Resources Science and Technology, Beijing Normal University, Haidian District, Beijing 100875, China

**Keywords:** *Liposcelis bostrychophila*, *Curcuma wenyujin*, contact toxicity, fumigant, essential oil composition, 1,8-cineole, camphor

## Abstract

The aim of this research was to determine the chemical composition and insecticidal activity of the essential oil of *Curcuma wenyujin* Y.H. Chen et C. Ling rhizomes against the booklouse *Liposcelis bostrychophila *Badonnel and to isolate any insecticidal constituents from the essential oil. The essential oil of *C. wenyujin* rhizomes was obtained by hydrodistillation and analyzed by GC-MS. A total of 43 components of the essential oil were identified and the principal compounds in the essential oil were 1,8-cineole (15.26%), camphor (10.12%), germacrone (6.86%), β-elemene (6.33%), curzerene (6.70%), and β-elemenone (5.23%). followed by curzerenone (4.52%), curdione (4.45%) and linalool (4.43%). Based on bioactivity-guided fractionation, the two main active constituents were isolated from the essential oil and identified as 1,8-cineole and camphor. The essential oil of *C. wenyujin* rhizomes exhibited contact toxicity against *L. bostrychophila* with an LD_50_ value of 208.85 µg/cm^2^. Camphor (LD_50_ = 207.26 µg/cm^2^) exhibited stronger contact toxicity than 1,8-cineole (LD_50_ = 1048.75 µg/cm^2^) against booklouse. The essential oil of *C. wenyujin* (LC_50_ = 2.76 mg/L air) also possessed fumigant toxicity against *L. bostrychophila*, while the two constituents, camphor and 1,8-cineole had LC_50_ values of 1.03 mg/L air and 1.13 mg/L air, respectively. The results indicate that the essential oil of *C. wenyujin* rhizomes and its constituent compounds have potential for development as natural insecticides or fumigants for control of insects in stored grains.

## 1. Introduction

The booklouse *Liposcelis bostrychophila* Badonnel (Psocoptera: Liposcelididae) has a worldwide distribution and is commonly found in various processed and unprocessed dry foods in households, granaries, and warehouses [1]. These are tiny (approximately 1 mm in length), wingless, light brown insects. Psocids used to be considered as nuisance pests rather than a cause of losses to stored commodities [2]. They were regarded as secondary pests, often overlooked due to their small size and the existence of other more damaging primary pests (e.g., maize weevils *Sitophilus zeamais*, rice weevils *S. oryzae* and lesser grain borer, *Rhyzopertha dominica*) in cereal grains [2]. However, currently, psocids are perhaps the most important category of emerging pests in stored grains and related commodities due to their small size and resistance to chemicals [2,3,4]. Infestations of stored product insects currently are controlled by fumigation or insecticidal treatment of commodities and surfaces [5]. However, many problems are associated with these chemicals, such as the development of resistance, toxic residues in food, workers’ safety, and high cost of procurement [5,6]. These problems have necessitated a search for alternative eco-friendly insect pest control methods [6]. The use of essential oils or their constituents with low mammalian toxicity can effectively prevent and/or suppress insect pest especially in storage [7]. Investigations in several countries confirm that some plant essential oils not only repel insects, but possess contact and fumigant toxicity against stored product pests as well as exhibiting feeding inhibition or harmful effects on the reproductive system of insects [8]. Essential oils from many plants including medicinal herbs, spices and fruits have been evaluated with success for insecticidal activity against stored product insects/mites, in some cases, have been proven more effective than traditionally used organophosphorus pesticides [9,10,11,12,13,14,15,16,17].

During a screening program for new agrochemicals from Chinese medicinal herbs and wild plants, the essential oil of *Curcuma wenyujin* Y.H. Chen et C. Ling (Zingiberaceae) rhizomes was found to possess strong insecticidal toxicity against the booklouse (*L. bostrychophila*). *Curcuma wenyujin* is an herbaceous perennial plant distributed mainly in Guangdong, Guangxi, and Zhejiang Provinces of China and has been cultivated for medicinal purposes [18], as its roots and rhizomes have been employed in traditional Chinese medicines in different medicinal agents, known as “Radix curcuma” and “Rhizoma wenyujin concisa”, respectively [19]. They are famous for their actions to eliminate blood stasis, stimulate menstrual discharge and relieve pain [19]. The chemical composition of the essential oil of *C. wenyujin* has been widely studied [20,21,22,23]. However, a literature survey has shown that there is no report on insecticidal activity of the essential oil of *C. wenyujin*. Thus we decided to investigate the chemical constituents and insecticidal activities of the essential oil of *C. wenyujin* rhizomes against a grain storage insect for the first time and to isolate any active constituent compounds from the essential oil.

## 2. Results and Discussion

### 2.1. Essential Oil Chemical Composition

The yield ofyellow essential oil of *C. wenyujin* rhizomes was 2.11% (v/w) and the density of the concentrated essential oil was determined as 0.88 g/mL. A total of 43 components of the essential oil of *C. wenyujin* were identified, accounting for 96.85% of the total oil (Table 1). The principal compounds inthe essential oil were 1,8-cineole (15.26%), camphor (10.12%), germacrone (6.86%), β-elemene (6.33%), curzerene (6.70%), β-elemenone (5.23%), curzerenone (4.52%), curdione (4.45%) and linalool (4.43%). Monoterpenoids represented 20 of the 43 compounds, corresponding to 50.31% of the whole oil, while 21 of the 43 constituents were sesquiterpenoids (45.41% of the crude essential oil). The chemical composition of the essential oil of *C. wenyujin* rhizomes in the present study was different from that reported in previous studies. For example, curdione (22.04%), curzerenone (18.0%), curzerene (15.85%), and germacrone (9.32%) were the main constituents in the essential oil of *C. wenyujin *rhizomes harvested from Zhejiang Province in the fall and winter [20]. However, the major components in the essential oil of *C. wenyujin *rhizomes, also collected from Zhejiang Province in June, were 1,8-cineole (30.63%), β-selinene (7.32%), α-copaene (6.65%), fenchol (6.56%), β-pinene (4.96%), and camphor (4.62%) [21]. The essential oil of *C. wenyujin *rhizomes purchased from a local Chinese herbs market contained germacrone (9.07%), curcumenol (8.53%), isocurcumenol (7.48%), *ar*-zingiberone (5.06%) and curzerenone (4.98%) [22]. Seasonal variations in chemical composition of the essential oil derived from *C. wenyujin *rhizomes have been also observed [20]. The above findings suggested that there are great variations in the chemical composition of the essential oil of *C. wenyujin *rhizomes. Thus, further studies on plant cultivation and essential oil standardization because chemical composition of the essential oil of *C. wenyujin *rhizomes varies greatly with the plant population as well as harvest time.

**Table 1:** Chemical constituents of the essential oil derived from *Curcuma wenyujin *rhizomes.

	RI *	Compound	Composition (%)
1	926	tricyclene	0.06
2	929	α-thujene	0.15
3	933	α-pinene	0.94
4	954	camphene	1.82
5	974	β-pinene	1.18
6	991	β-myrcene	0.49
7	1002	(+)-4-carene	0.39
8	1031	1,8-cineol	15.26
9	1059	γ-terpinene	0.14
10	1091	2-nonanone	0.84
11	1094	linalool	4.43
12	1146	camphor	10.12
13	1162	isoborneol	1.23
14	1167	borneol	3.83
15	1179	4-terpineol	3.02
16	1182	*p*-cymen-8-ol	2.32
17	1189	α-terpineol	3.19
18	1204	verbenone	0.35
19	1217	*trans*-carveol	0.34
20	1238	carvone	0.18
21	1277	isobornyl acetate	0.87
22	1293	2-undecanone	0.29
23	1334	δ-elemene	0.55
24	1375	α-copaene	1.76
25	1385	β-bourbonene	0.63
26	1388	β-patchoulene	0.38
27	1394	β-elemene	6.33
28	1420	β-caryophyllene	0.17
29	1433	γ-elemene	0.22
30	1498	α-muurolene	0.23
31	1454	α-caryophyllene	0.45
32	1485	germacrene D	0.66
33	1494	α-selinene	1.11
34	1498	curzerene	6.70
35	1502	β-guaiene	0.48
36	1523	δ-cadinene	0.66
37	1578	spathulenol	2.53
38	1583	caryophyllene oxide	0.72
39	1589	β-elemenone	5.23
40	1648	β-eudesmol	0.77
41	1680	curzerenone	4.52
42	1688	germacrone	6.86
43	1893	curdione	4.45
		Total identified	96.85
		Monoterpenoids	50.31
		Sesquiterpenoids	45.41
		Others	1.13

* RI, retention index as determined on a HP-5MS column using the homologous series of *n*-hydrocarbons.

### 2.2. Insecticidial Activities

The essential oil of *C. wenyujin* rhizomes exhibited contact toxicity against *L. bostrychophila* with an LD_50_ value of 208.85 µg/cm^2^ (Table 2). When compared with the positive control pyrethrum extract (LD_50_ = 18.99 µg/cm^2^), the essential oil was 11 times less toxic to *L. bostrychophila*. Two of the constituent compounds, camphor and 1,8-cineole exhibited contact toxicity against booklice with LD_50_ values of 207.26 µg/cm^2^ and 1048.75 µg/cm^2^, respectively (Table 2). Camphor possessed almost five times more toxicity than 1,8-cineole against booklice. It is thus suggested that camphor is a major contributor to the insecticidal (contact) activity of the essential oil. However, compared with pyrethrum extract (positive control), camphor and 1,8-cineole showed five times and 55 times less toxicity against booklice.

**Table 2:** Contact toxicity and fumigant toxicity of the essential oil of *Curcuma wenyujin* rhizomes and its constituents against *Liposcelis bostrychophila*.

	Treatment	LD_50 _LC_50_	95% FL *	Slope ± SE	Chi square (χ^2^)
Contact Toxicity (μg/cm^2^)	*C. wenyujin*	208.85	189.48–227.16	5.01 ± 0.63	35.84
	1.8-Cineol	1048.74	1021.95–1096.85	9.50 ± 0.91	11.76
	Camphor	207.26	199.78–214.99	13.81 ± 1.47	15.87
	Pyrethrum extract	18.99	17.56–20.06	7.64 ± 1.05	7.35
Fumigant (mg/L air)	*C.wenyujin*	2.76	1.95–3.67	2.57 ± 0.35	27.37
	1.8-Cineol	1.13	1.01–1.21	5.80 ± 0.67	13.57
	Camphor	1.03	0.94–1.11	6.13 ± 0.63	12.19
	Dichlorvos	1.35 × 10^−3^	1.25-1.47 × 10^−3^	6.87 ± 0.77	5.43

* Fiducial limits

**Figure 1:**
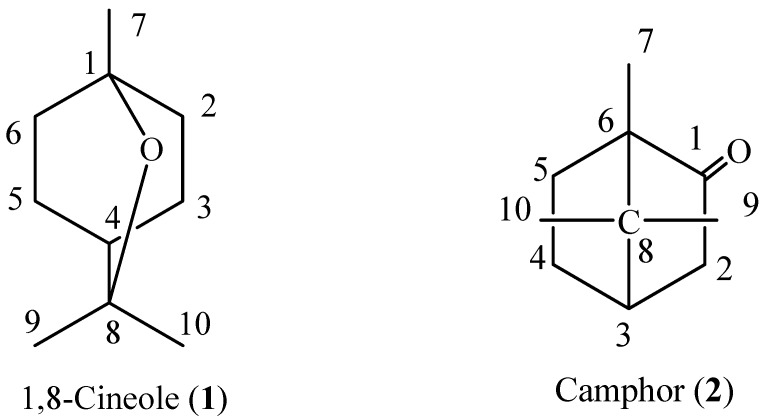
Constituent compounds isolated from the essential oil of *Curcuma wenyujin* rhizomes.

Camphor and 1,8-cineole (Figure 1) possessed fumigant toxicity against *L. bostrychophila* adults with LC_50_ values of 1.03 mg/L air and 1.13 mg/L air, respectively, while the crude essential oil of *C. wenyujin* rhizomes showed an LC_50_ value of 2.76 mg/L air (Table 2). 

Compared with the positive control, dichlorvos (LC_50_ = 1.35 μg/L air), the two isolated constituent compounds exhibited almost 762 and 837 times less toxicity to *L. bostrychophila*, respectively. Camphor exhibits repellency against clothes moths like *Tineola bisselliella* and has been used as a less-toxic natural ingredient (compared with synthetic repellents, e.g., naphthalene and 1,4-dichlorobenzene) in mothballs [24]. In the previous reports, camphor has been demonstrated to exhibit contact and fumigant toxicity against several stored products insects [25,26]. Camphor also exhibited insecticidal activity against many important pest insects/mites, e.g., the cotton leafworm, *Spodoptera littoralis* (the 3rd larvae stage, LC_50_ = 5.61 mg/L) [27], larvae of *Pseudaletia unipuncta*(the 2nd larvae, LD_50_ = 189.4 μg/larva) and *Trichoplusia ni* (the 3rd larvae, LD_50_ = 233.9 μg/larva) [28], fruitflies, *Drosophila melanogaster* (LC_50_ = 4.82 mL/L air) and *Bactrocera oleae* (LC_50_ = 1.45 mL/L air) [29], adults of German cockroaches, *Blattella germanica* (LD_50_ = 0.10–0.14 mg/cm^2^) [30], *Pediculus humanus capitis* (a 12 h LD_50_ = 5.4 mg/cm^2^) [31], mosquitoes, *Aedes aegypti* [32] and copra mite, *Tyrophagus putrescentiae* [33] as well as house dust mite, *Dermatophagoides pteronyssinus * [34]. Moreover, in the previous studies, 1,8-cineole was found to exhibit fumigant toxicity against red flour beetles, *Tribolium castaneum* adult with LC_50_ = 41 μL/L air [35], 15.3 μL/L air [36], and 1.52 mg/L air [37]. It also possess fumigant toxicity against several other stored product insects and cockroaches as well as mosquitoes [32,38,39,40], e.g., the rice weevil (*S. oryzae*) (LC_50_ = 22.8 μL/L air), and the lesser grain borer (*Rhyzopertha dominica*) (LC_50_ = 9.5 μL/L air) [36]. It seems that the mentioned stored product insects were more tolerant than booklice to 1,8-cineole. Moreover, 1,8-cineole was also found to possess fumigant activity against agricultural important pests, e.g., the cotton leafworm, *S. littoralis* (the third larvae, LC_50_ = 4.34 mg/L) [27] and fruitflies, *D. melanogaster* (LC_50_ = 1.19 mL/L air) and *B. oleae* (LC_50_ = 0.50 mL/L air) [29]. The above findings suggest that that insecticidal activity, especially the fumigant activity of the essential oil of *C. wenyujin* rhizomes and its two constituent compounds, against the booklouse is quite promising. As currently used fumigants are synthetic insecticides and the most effective fumigants (e.g., phosphine and MeBr) are also highly toxic to humans and other non-target organisms, the essential oil of *C. wenyujin* rhizomes and its two constituent compounds show potential to be developed as possible natural fumigants/insecticides for the control of *L. bostrychophila*.

In Traditional Chinese Medicine, *C. wenyujin* rhizomes are shown to possess many pharmacological actions such as anti-tumor, anti-early-pregnancy, antibacterial, leukocytes-increasing, body-rheography-improving, acute-kidney-failure alleviating, platelet-aggregation-inhibiting, anti-thrombosis and anti-inflammatory activities were demonstrated [19]. It seems that this medicinal herb is quite safe for human consumption because it has been used as a medicinal herb for hundreds of years. However, no experimental data about the safety of this herb is available so far. The isolated constituent camphor is reported to have oral LD_50_ value of more than 5 g/kg body weight in rats and 1.31 g/kg body weight in mice [41]. In humans the signs of camphor intoxication include central nervous stimulation, oral and gastric irritation, nausea and vomiting, excitement, hallucinations, delirium, muscular excitability, tremors, convulsions and urinary retention [41]. The reported acute toxicity data of camphor on adults and children arise mostly from accidental ingestion of camphor-containing medications. The probable lethal oral dose has been reported to be in the range of 50 to 500 mg/kg body weight. No acute toxicity of camphor was reported after doses lower than 2 mg/kg body weight [42]. Thus, this compound is relatively safe for human consumption. WHO experts concluded that it would not raise a safety concern at current estimated intakes of camphor as flavouring substance of 58 μg/day in Europe and 396 μg/day in the USA [42]. The another isolated constituent 1,8-cineole is reported to have oral LD_50_ of 2.48g/kg body weight for rats and it is classified as a reproductive toxin for females and a suspect reproductive toxin for males [43]. Thus, to develop a practical application for the essential oil and the isolated constituents as novel fumigants/insecticides, further research into the safety of the essential oil/compounds to humans is needed. Additional studies on the development of formulations are also necessary to improve the efficacy and stability and to reduce cost.

## 3. Experimental

### 3.1. Plant Material and Essential Oil Extraction

The fresh rhizomes of *C. wenyujin* (10 kg) were harvested at August 2011 from Ruian Country (27.78° N latitude and 120.63° E longitude, Zhejiang Province, China). The plant was identified by Dr. Liu, QR (College of Life Sciences, Beijing Normal University, Beijing, China) and a voucher specimen (CMH-Jianghuang-Zhengjiang-2011-08) was deposited in the museum of Department of Entomology, China Agricultural University. The sample was air-dried and ground to a powder using a grinding mill (Retsch Muhle, Germany). The powder was subjected to hydrodistillation using a modified Clevenger-type apparatus for 6 h and extracted with *n*-hexane. Anhydrous sodium sulphate was used to remove water after extraction. The essential oil was stored in airtight containers in a refrigerator at 4°C for subsequent experiments.

### 3.2. Insects

Booklice, *L. bostrychophila*, were obtained from laboratory cultures in the dark in incubators at 28–30 °C and 70%–80% relative humidity and was reared on a 1:1:1 mixture, by mass, of milk powder, active yeast, and flour. All the containers housing insects and the Petri dishes used in experiments were made escape proof with a coating of polytetrafluoroethylene (Fluon®, Blades Biological, Cowden Edenbridge, Kent, UK). Laboratory bioassays were done within one week after adult collections.

### 3.3. Gas Chromatography-Mass Spectrometry

Components of the essential oil of *C. wenyujin* rhizomes were separated and identified by gas chromatography-flame ionization detection (GC-FID) and gas chromatography–mass spectrometry (GC–MS) Agilent 6890N gas chromatography connected to an Agilent 5973N mass selective detector. The same column and analysis conditions were used for both GC-FID and GC-MS. They were equipped with a flame ionization detector and capillary column with HP-5MS (30 m × 0.25 mm × 0.25 μm). The GC settings were as follows: the initial oven temperature was held at 60 °C for 1 min and ramped at 10 °C min^−1^ to 180 °C where it was held for 1 min, and then ramped at 20 °C min^−1^ to 280 °C and held there for 15 min. The injector temperature was maintained at 270 °C. The samples (1 μL) were injected neat, with a split ratio of 1:10. The carrier gas was helium at flow rate of 1.0 mL min^−1^. Spectra were scanned from 20 to 550 *m/z* at 2 scans s^−1^. Most constituents were identified by gas chromatography by comparison of their retention indices with those of the literature or with those of authentic compounds available in our laboratories. The retention indices were determined in relation to a homologous series of *n*-alkanes (C_8_–C_24_) under the same operating conditions. Further identification was made by comparison of their mass spectra with those stored in NIST 05 (Standard Reference Data, Gaithersburg, MD, USA) and Wiley 275 libraries (Wiley, New York, NY, USA) or with mass spectra from the literature [44]. Component relative percentages were calculated based on GC peak areas without using correction factors.

### 3.4. Contact Toxicity with Treated Filter Paper

Range-finding studies were run to determine the appropriate testing concentrations of the essential oil of *C. wenyujin* and pure compounds. The essential oil and compound were diluted in acetone. The filter paper with 3.5 cm in diameter (Whatman) was treated with 150 μL of the solution. Then the filter paper after treated with solid glue (Glue Stick, Jong Ie Nara Co., Ltd. Hong Kong) was placed in a Petri dish (3.5 cm in diameter) and 10 booklice were put on the filter paper by using a hair brush. The plastic cover with holes was put and all the Petri dishes were kept in incubators at 27–29 °C, 70%–80% r.h. for 24 h. Acetone was used as controls and pyrethrum extract was used as a positive control. Five concentrations and five replicates of each concentration were used in all treatments and controls. Mortality of insects was observed and the observed data were corrected for control mortality using Abbott’s formula. The results from all replicates were subjected to probit analysis using the PriProbit Program V1.6.3 to determine LC_50_ values [45]. Pyrethrum extract (25% pyrethrine I and pyrethrine II) was purchased from Fluka Chemie (Buchs, Switzerland).

### 3.5. Fumigant Toxicity

Range-finding studies were run to determine the appropriate testing concentrations of the pure compounds and *C. wenyujin* essential oil. A filter paper strip (3.5 cm × 1.5 cm) treated with 10 μL of an appropriate concentration of test essential oil/compound in acetone. The impregnated filter paper was then placed in the bottom cover of glass bottle of 250 mL. The insects, 10 adults in a small glass bottle (8 mL), were exposed for 24 h and each concentration with five replicates. All the treatments were replicated five times. Acetone was used as controls and dichlorvos was used as a positive control. The observed mortality data were corrected for control mortality using Abbott’s formula. The LC_50_ values were calculated by using Probit analysis [45]. Positive control, dichlorvos (99.9%) was purchased from Aladdin Reagent Company (Shanghai, China).

### 3.6. Bioassay-Directed Fractionation

The crude essential oil of *C. wenyujin* rhizomes (25 mL) was chromatographed on a silica gel (Merck 9385, 1,000 g) column (85 mm i.d., 850 mm length) by gradient elution with a mixture of solvents (*n*-hexane, *n*-hexane-ethyl acetate). Fractions (500 mL each) were collected and concentrated at 40 °C, and similar fractions according to thin layer chromatography (TLC) profiles were combined to yield 15 fractions. Fractions (3–5, 8) that possessed contact toxicity, with similar TLC profiles, were pooled and further purified by preparative silica gel column chromatography (PTLC) until to obtain the pure compound for determining structure as 1,8-cineole (0.6 g) and camphor (0.8 g). The structure of the compounds was elucidated based on high-resolution electron impact mass spectrometry and nuclear magnetic resonance. ^1^H and ^13^C-NMR spectra were recorded on Bruker ACF300 and AMX500 (500 MHz (^1^H)) instruments using CDCl_3_ as the solvent with TMS as internal standard. EIMS were determined on a ThermoQuest Trace 2000 mass spectrometer at 70 eV (probe).

*1,8-Cineole *(Eucalyptol, **1**), colorless oil. MS *m/z* (%): 154 (24), 111 (29), 108 (36), 96 (23), 93 (56), 84 (38), 81 (56), 71 (47), 69 (40), 68 (38), 67 (24), 55 (33), 43 (100), 41 (33), 39 (19). C_10_H_18_O. ^1^HNMR (500 Hz, CDCl_3_) δ: 1.05 (3H, 7-CH_3_), 1.24 (6H, 9, 10-CH_3_), 1.41 (1H, 4-H), 1.50 (4H, Ph-H), 1.66 (2H, Ph-H), 2.02 (2H, Ph-H). ^13^CNMR (CDCl_3_) δ: 76.8 (C-8), 72.7 (C-1), 39.6 (C-4), 37.3 (C-2, C-6), 28.9 (C-9, 10), 25.4 (C-7), 24.2 (C-3, 5). The spectral data matched with the previous reports [46,47].

*Camphor* (**2**), white solid. MS *m/z* (%): 152 (30), 110 (14), 109 (30), 108 (43), 95 (100), 83 (34), 81 (72), 69 (34), 67 (18), 55 (29), 41 (44). C_10_H_16_O. ^1^H-NMR (500 Hz, CDCl_3_) δ: 2.36 (1H, H-2), 2.09 (1H, H-3), 1.96 (1H, H-4), 1.85 (1H, H-2), 1.68 (1H, H-5), 1.37 (2H, H-4, 5), 0.96 (3H, 9-CH_3_), 0.92 (3H, 7-CH_3_), 0.84 (3H, 10-CH_3_). ^13^C-NMR (CDCl_3_) δ: 219.0 (C-1), 57.7 (C-6), 46.8 (C-2), 43.3 (C-8), 40.1 (C-3), 30.0 (C-5), 27.1 (C-4), 19.8 (C-9) 19.1 (C-10), 9.3 (C-7). The data matched with the previous reports [47,48,49].

## 4. Conclusions

The study indicates that the essential oil of *C. wenyujin* rhizomes and its constituent compounds camphor and 1,8-cineols have potential for development into natural insecticides/fumigants for control of insects in stored grains.
